# Supplementary material for: Effect of low-intensity focused ultrasound therapy on postpartum uterine involution in puerperal women: A randomized controlled trial
Source: PLoS One. 2024 Apr 30;19(4):e0301825. doi: 10.1371/journal.pone.0301825 (PMC11060566; doi:10.1371/journal.pone.0301825)
Supplement: S1 Protocol — (DOCX) [file pone.0301825.s001.docx]

Clinical trial protocol of ultrasound postpartum rehabilitation therapy instrument

Name of the experimental medical device: ultrasound postpartum rehabilitation treatment instrument

Model and specification: TY-200A

Management category of investigational medical devices : Class II

The third type of medical device subject to clinical trial approval is : Yes 🞎 or No🞎

Solution version number and date: Version 4.0 /20190326

Clinical trial institution: West China Second Hospital of Sichuan University

Principal investigator: Dongmei Wei

Sponsor: Sichuan Taiyou Technology Co., LTD

Agent: None

filling explanation:

1 . For multi-center clinical trials, the clinical trial institution on the cover only fills in the lead unit, and the other institutions are listed in the protocol content.

2. For multi-center clinical trials, the investigator on the cover shall fill in and coordinate the investigator.

Clinical trial protocol of ultrasound postpartum rehabilitation instrument / version 4. 0 / 2O19O326

Sponsor information

| Company name | Sichuan Taiyou Technology Co., LTD | | | |
| --- | --- | --- | --- | --- |
| project leader | Li Dong | | | |
| Contact address | No. 1, 2, 3, 6th Floor, Unit 7, Block D, Xibuzhigu, No. 166, Wuxing 4th Road, Wuhou District, Chengdu City, Sichuan Province | | Postcode | 610213 |
| telephone |  |  | Cell ph |  |
| portraiture |  | | E-mail |  |

Statistical analysis unit

| Company name | | Research Center of Evidence-Based Medicine and Epidemiology, West China Hospital, Sichuan University | | |
| --- | --- | --- | --- | --- |
| Actuary | Deying Tang | |  |  |
| Contact address | No.37, Guoxue Lane, Wuhou District, Chengdu | | Postcode | 610041 |
| telephone |  | | Cell phone |  |
| telephone |  | | E-mail |  |

List of researchers

| Principal  investigator | Wei Dongmei, West China Second Hospital of Sichuan University | |
| --- | --- | --- |
| Central number | 01 | |
| address | No.20, Section 3, Renmin South portra Road | Postcode 610047 |
| telephone |  |  |

| Scheme  number | SCTYCH-2 |
| --- | --- |
| scenario  name | Clinical trial of ultrasound postpartum rehabilitation therapy instrument |
| version  number | Version 4.0 |
| The  applicant | Sichuan Taiyou Technology Co., LTD |
| Clinical  trial score | Medical device class II |
| indication | This therapeutic instrument is suitable for the rehabilitation of the postpartum uterus and breast |
| test  objective | Observe the effect of the ultrasound postpartum rehabilitation  instrument on postpartum uterine recovery and breast pain, and |
| experiment design | This trial uses a randomized, parallel-controlled, single-blind design. The treatment group added ultrasound therapy to the conventional treatment, and the control group added device sham therapy to the conventional treatment. The comparison of the two data  proves the effectiveness and safety of ultrasound postpartum |
| Total  number of cases | A total of 176 cases were recorded. |
| Number of research centers | Three centers |
| The study period | It is expected to start from January 1,2019 |

Abstract

| Case  selection  criteria  standard | This validation is for healthy women, in order to exclude the factors affecting uterine contraction and lochia discharge as far as possible, cesarean section and vaginal delivery inclusion and exclusion criteria.  1.Screening criteria for cesarean section:  (1) Inclusion criteria for cesarean section:  No serious complications and comorbidities during antenatal  examination:  Normal term 37-42 weeks of elective surgery, postpartum voluntary  breastfeeding maternal ;  Signed the informed consent form.  (2)Exclusion criteria for women delivered by cesarean section:  Severe preeclampsia : intrahepatic cholestasis in pregnancy:  Twin twins, macrosomia, too much amniotic fluid ;  Coagulation dysfunction APTT, time greater than 40s, platelet count less than 80× 10^7^L:  Other systemic diseases that are uncontrolled ;  Metal implants are present in the body:  Allergy to an ultrasound coupling agent :  Other diseases that the researchers considered unsuitable for  inclusion.  2. Screening criteria for vaginal delivery:  (1)Inclusion criteria for women with vaginal birth :  There were no serious complications and comorbidities in the  prenatal examination;  Normal term 37-42 weeks of birth, voluntary breastfeeding  postpartum women  Signed the informed consent form.  (2)Exclusion criteria for women delivered by cesarean section:  Severe preeclampsia : intrahepatic cholestasis in pregnancy:  Twin twins, macrosomia, too much amniotic fluid ;  Coagulation dysfunction APTT, time greater than 40s, platelet count less than 80× 10^7^L:  Other systemic diseases that are uncontrolled ;  Metal implants are present in the body:  Allergy to an ultrasound coupling agent :  Other diseases that the researchers considered unsuitable for  inclusion. |
| --- | --- |

Clinical trial protocol of ultrasound rehabilitation / version 4. 0 / 20190326

| stages of  research | Screening period  1.Pregnant women screened for labor, sign informed consent, and meet  their inclusion / exclusion criteria will be randomized.   1. The investigator recorded the patient's demographic data and related medical history, reviewed the enrollment and exclusion criteria one by one, and informed the patient of the pain assessment method, the nature of lochia and lochia amount, and the follow-up   time.  stage of therapy  1.The investigator recorded the duration of treatment and  treatment of patients in the trial and control groups.  2. Observe and record the height of the posterior fundus drop,  lochia changes, and breast swelling pain score before treatment.  3. Record of adverse events.  follow-up period  1.The researcher examined the filling of medical records  2.The subjects were followed up regularly and the end time of lochia was recorded. |
| --- | --- |
| Efficacy  indicators | Main efficacy indicators  Uterus fundus index:(uterus fundus position before first treatment-uterus fundus position after 3 treatment) / (uterus fundus position before first treatment)×100%, calculate the uterine floor drop index, and evaluate the effect of the control group. If there was a significant statistical difference in the uterine floor drop  index, it was judged to be effective.  Effective rate of breast pain relief: VAS score was used to evaluate the degree of maternal breast pain, reducing the degree of breast pain by> 30% compared with baseline. Effective breast number / total trial breast number ×100%. There was a significant statistical difference in the response rate of breast pain relief in the ultrasound treatment group compared with the control group.  Secondary efficacy indicators  Termination time of lochia: The end time of lochia under ultrasound treatment for vaginal delivery and cesarean section and the control group were counted respectively, and the influence of ultrasound treatment on the end time of lochia was evaluated. If there was a significant statistical difference, it was judged to be effective.  The poor involution rate : The number of people whose lochia ended for more than 42 days was counted and the percentage was calculated. The defective rate of uterine rejuvenation between the two groups was compared. If there was a significant statistical difference in the defective rate of uterine rejuvenation, it was judged to be effective. |

Clinical trial protocol of ultrasound rehabilitation / version 4. 0 / 20190326

| Safety  indicators | Adverse events (clinical adverse events). |
| --- | --- |
| statistica l analysis | For group comparison of efficacy indicators, significance level =0. 05 (unilateral), other hypothesis tests that can be involved were  two-sided, and PW 0.05 was considered statistically significant. |

**Flow chart of study**

| Clinical trial phase | Baseline data | [Treatment period](javascript:;) | [Follow-up period](javascript:;) |
| --- | --- | --- | --- |
| [View window](javascript:;) | The -7 ~ 1 day | 2 ~ 4 days [postpartum](javascript:;) | 7±2、21±2、35±2、42±2 days [postpartum](javascript:;) |
| Informed Consent | √ |  |  |
| Demographic Data | √ |  |  |
| History and physical examination | √ | √ | √ |
| Inclusion/exclusion criteria | √ |  |  |
| Laboratory tests(routine blood tests;liver/kidney function test) | √ |  |  |
| ECG | √ |  |  |
| Uterine fundus height | √ | √ |  |
| Duration of postpartum lochia |  | √ | √ |
| [Breast pain](javascript:;)(VAS) | √ | √ |  |
| Treatment parameters |  | √ |  |
| [Adverse event](javascript:;)s |  | √ | √ |

content

[Clinical trial protocol of ultrasound postpartum rehabilitation therapy instrument 1](#_Toc18882)

[List of researchers 3](#_Toc17360)

[Flow chart of study 9](#_Toc18421)

[Abbreviation 11](#_Toc28745)

[1. Purpose and content of the clinical trial 12](#_Toc30198)

[(1) purpose 12](#_Toc21027)

[(2) content 12](#_Toc31337)

[2. Background information of the clinical trials 12](#_Toc32225)

[3 Characteristics, structure and composition, working principle, mechanism of action and test scope of the products 15](#_Toc9869)

[4 Indications, contraindications and precautions of the product 18](#_Toc24031)

[5. overall design 19](#_Toc30810)

[6. statistical considerations 30](#_Toc24670)

[7. data management 32](#_Toc23275)

[8. feasibility analysis 33](#_Toc21599)

[9. Quality control of clinical trials 33](#_Toc19853)

[10. ethical issues and informed consent forms for clinical trials 35](#_Toc5949)

[11 Provisions for the reporting of adverse events and device defects 36](#_Toc12561)

[12 The deviation of the clinical trial protocol and the amendment of the clinical trial protocol 40](#_Toc3093)

[13 Finance and Insurance 40](#_Toc836)

[14 Principles of confidentiality 41](#_Toc10498)

[15 Agreement on the publication of the test results 41](#_Toc5845)

[16 Responsibilities of all parties 42](#_Toc28005)

[17 Investigator Statement 43](#_Toc20919)

Abbreviation

AE adverse event

CRA

CRF

ECG

SAE

SD

SFDA

VAS

Clinical Research Associate

Case report form

electrocardiogram

Serious adverse events

standard deviations

The State Food and Drug

Administration

visual analogue scales

### 1. Purpose and content of the clinical trial

1. purpose

Observe the effect of ultrasound postpartum rehabilitation instrument on postpartum uterine recovery and relieving breast pain, and evaluate the safety and effectiveness of ultrasound postpartum rehabilitation instrument.

1. content

Effectiveness study: observe the effect of ultrasound postpartum rehabilitation treatment instrument on uterine floor drop index,

termination time of lochia and breast pain in postpartum patients.

Safety study: Observe the clinical adverse reactions during ultrasound postpartum rehabilitation device and study the safety of the device.

### 2. Background information of the clinical trials

(1) After 6 weeks postpartum, the uterus still fails to recover to a non-pregnant state, which is called postpartum uterine recovery. The main clinical manifestations are continuous vaginal bleeding or repeated intermittent vaginal bleeding and postpartum abdominal pain.

In recent years, with the increasing rate of cesarean section, the cases of prolonged lochia after cesarean section are increasing. In the early stage of cesarean section, the uterine  recover rate is slow, and the overall  recover rate is more uniform; the early uterine recovery after vaginal delivery is fast and slow. In clinic, most women need different treatments to promote uterine recovery after delivery. Uterine smooth muscle contraction is a necessary condition for uterine involution, 70%- 80% of postpartum bleeding is caused by fatigue of uterine contraction, so postpartum strengthening uterine contraction is an effective measure to promote postpartum uterine involution. For the traditional drugs and methods to promote uterine contraction, there are various means, although they all have certain efficacy, but each has its own advantages and disadvantages, and it is difficult to achieve satisfactory results.

For example:

1. Injection of oxytocin: In clinical treatment, it is found that oxytocin will cause postpartum uterine tonic contraction, which can aggravate the pain of maternal uterine contraction.

2 . Oral drugs such as yi mu cao: They help with uterine contraction, but the effect is slow.

3.Traditional Chinese medicine massage: postpartum massage for the mechanical and physical effect of the hand. One is to push the uterus; the other is the rhythm of massage and the rhythmic contraction of the uterus; the other is the regulation of the meridian and the rapid recovery.

4. Ultrasound therapy has been applied in chronic soft tissue injury for more than 60 years. Due to its non-invasive, no side effects, safety and simplicity, it has attracted more and more researchers' attention, and various ultrasound therapy instruments at home and abroad have emerged. Uterine smooth muscle also belongs to soft tissue, and the report of the effect of ultrasound on uterine smooth muscle has also attracted more and more attention from researchers. According to the literature reports at home and abroad, the therapeutic effect of ultrasound on soft tissue injury has been unanimously affirmed, and there is a clear effect on the chronic soft tissue injury of smooth muscle. Due to the lack of high frequency ultrasonic penetration depth, the therapeutic energy can not reach the lesion site: focus ultrasound, the acoustic is the ultrasonic therapeutic energy at one point, the treatment range is limited and the energy is too large: low frequency ultrasound, sound has good tissue penetration,

localization and energy deposition, and can reach the therapeutic dose; low frequency, rate convergence ultrasound using the characteristics of low frequency ultrasound and energy convergence to the treatment site, can achieve the therapeutic effect and the treatment range is

unrestricted.

Wang Qianqian et al. ⑴ studied 73 patients into trial group (ultrasound treatment group) and control group (ultrasound false treatment) by random method. The study results showed that low-intensity focused ultrasound treatment could promote postpartum uterine recovery

without no obvious adverse reactions.

(2) With the opening of the national two-child policy and the increase of life pressure, women often choose to work as soon as possible after giving birth, on the one hand, to relieve the pressure of the family, on the other hand, they worry that postpartum breastfeeding will cause breast sagging. So most of the women do not feed and do not timely return milk is very easy to cause breast pain; for postpartum breastfeeding women if prenatal in a state of high life pressure and personal physical factors will cause many mothers will postpartum milk deficiency, postpartum milk deficiency will lead to the reduction of breastfeeding rate, so it is important to do a good job of postpartum breast care.

The clinical care for breast pain is mainly to use hot compress and massage method to dredge the breast tube to promote milk secretion. If the massage technique will only increase the maternal breast pain, therefore more reluctant to massage breast and feeding the baby, thus forming a vicious circle, when the breast pain can not be timely relief serious can cause milk, aden itis; for milk deficiency mainly through tonic methods such as carp soup to promote milk secretion, but the effect of tonic is slow and maternal digestion and absorption. If the soup is too greasy and the maternal digestion and absorption ability is poor, the milk is not obvious and easy to cause fat accumulation in all parts of the maternal body; the baby contains too much fat in the milk and cannot digestion, resulting in diarrhea.

At present, ultrasound treatment can be used for breast care. Low intensity convergence ultrasound has good tissue penetration, localization and energy deposition performance. The mechanical effect of ultrasound is the basic primary effect of ultrasound, which can enhance cell membrane permeability, improve metabolism, promote blood circulation, stimulate nervous system and group, and current can excite smooth muscle and adjust nerve, with high carrier frequency, small skin irritation, large electrical flow and deep effect. All of these are conducive to the discharge of milk, and the exclusion of milk can be stimulated.

The secretion of milk, so that the discharge of lactation and milk form a virtuous cycle, promote each other, so as to improve the rate of breastfeeding, reduce the small spoon, rubber nipple and other nipple illusion caused to the baby and reduce the lack of lactation.

Zhang Xueru et al. ⑵ used ultrasound prolactin in 312 cases of full-term delivery. The start time of lactation in the treatment group was more within 24h, which was significantly different compared with the control group (P <0.01). This study found that ultrasound promoted the secretion and discharge of milk but also stimulated uterine contraction and reduced vaginal bleeding.

### 3 Characteristics, structure and composition, working principle, mechanism of action and test scope of the products

(1) Product characteristics

The ultrasound postpartum rehabilitation therapy instrument is equipped with low-frequency convergence ultrasound technology.

Ultrasonic using its good tissue penetration, localization and energy deposition, energy convergence in treatment, to ensure that the ultrasound in the uterus, treatment of energy, ultrasonic mechanical effect can effectively stimulate human uterine smooth muscle rhythmic contraction, significantly relieve postpartum uterine contraction, lochia with long time and postpartum abdominal pain, and promote the uterine recovery.

For the ultrasound irradiation of breast parts, the mechanical effect, biochemical effect and thermal effect of ultrasound can achieve the effect of relieving the breast pain of patients.

(2) Product structure composition, working principle and mechanism of action

1. structural composition

As shown in Figure 1 below is the block diagram of the ultrasound postpartum rehabilitation therapy instrument."Processor" is the command center of the therapeutic instrument. When the "key" information is sent to the "processor", the processor will automatically identify the type of information and issue instructions to adjust the corresponding part. For example, if the "key" information adjusts the treatment time to a certain value, the "processor" will output instructions to the "timer",

so that the time of the "timer" is set on the predetermined value.


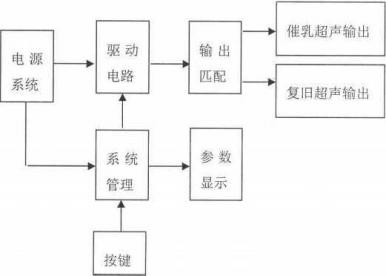


Figure 1 Structure composition block diagram

The operating parameters of the therapeutic device are displayed on the host panel

as follows: gear, time, position, etc.

The following figure shows the physical appearance of the ultrasound postpartum rehabilitation therapy instrument: the main machine includes the special cabinet for placing consumables, treatment head connection port and display window, mainly for control and parameter display functions. As shown in Figure 2.1, the treatment head connection of the main machine has two output: uterine recovery output interface and ultrasonic lactation output interface; the treatment head is as shown in Figure 2.2; the treatment head has two kinds: the handle is for uterine rehabilitation; the round head is for ultrasonic lactation; and the fixed device completes the treatment head to fit the ultrasonic transmitting end surface of the treatment head well and keep the position unchanged.

ma i n engine:


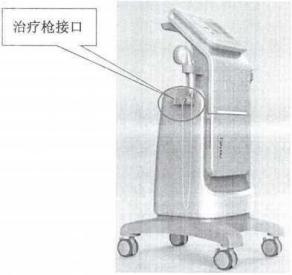


figure 2.1

Treatment components:


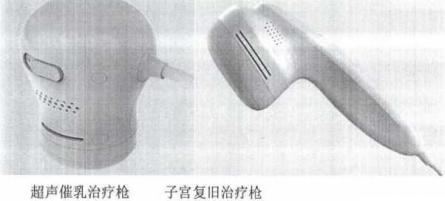


figure 2.2

2. operational principle

The mechanism of action of ultrasound mainly includes mechanical, thermal, and biochemical effects. Because ultrasonic wave is a mechanical wave, its vibration causes the particles in the human tissue to alternating compression and extension of positive pressure and negative pressure, and the resulting huge acceleration, so that the dissociation of ions in the liquid to obtain different motion speed. The ions with large

particles lag behind the ions with small particles, and the ions will move

relatively between each other, causing friction and forming energy. Ultrasonic vibration can cause the movement of substances in tissue cells, thus showing a fine massage effect: it can cause volume changes and produce cytoplasm flow. The oscillation, rotation and friction of cytoplasmic particles can stimulate the diffusion process of cell semi-permeable membrane, cause the change of diffusion speed and membrane permeability, promote the new generation, strengthen the blood and lymphatic circulation, improve the tissue nutrition, change the protein synthesis rate, and improve the tissue regeneration function. The mechanical effect of ultrasound changes the internal structure of the cell, leading to a series of functional changes of the cell, magnifying the physiological signals, strengthening the uterine contraction, and relieving the breast

swelling and pain.

### 4 Indications, contraindications and precautions of the product

(1) indication

This instrument is suitable for rehabilitation of postpartum uterus and breast.

(2) contraindication

1 . Placental residue, placental abruption, placenta previa;

2 . Coagulation dysfunction;

3. Severe preeclampsia;

4.Other systemic system diseases in which the condition is uncontrolled.

Possible adverse reactions or complications and their prevention and treatment:

1 . Skin burns: Due to inappropriate coupling or treatment of hair fever or local sensory disturbance, possible irritation of the skin in the treatment area, similar to burn-like adverse effects:

Ⅰ Burns can be left untreated.

ⅡBurn should pay attention to maintain the integrity of the skin, can be local burn ointment, conducive to healing.

2 Skin allergic reaction: the main manifestations is skin itching, red rash. Treatment should be stopped, avoid recontact, avoid scratching and scalding, or oral cetirizine, or external eudrow, which will generally disappear in 1 week.

Treatment of acid swelling: can tolerate the treatment area acid, pain is the normal response of treatment, such as file now can not tolerate the treatment area pain, generally do not do treatment, relief after rest.

As long as the instructions for use are followed, the above adverse

reactions will generally not occur in the course of treatment.

(3) Matters needing attention

1. Pay close attention to the maternal response during the treatment process, and adjust the movement speed at any time. Pay attention to the

coupling properties, drying and adhesion.

2. During the treatment and during the follow-up, the puerpera should be warned to use the same dose of related drugs to promote uterine recovery (oxytocin) according to the doctor's advice, and do not accept other related physical therapy affecting uterine rehabilitation (low-frequency electrical stimulation of pelvic floor recovery) or acupuncture treatment, so as not to affect the observation results.

### 5. overall design

(1) Test design

1. Test objective: To observe the effect of ultrasound postpartum rehabilitation treatment instrument on postpartum uterine recovery and relief of breast pain, and to evaluate the effectiveness and safety of

ultrasound postpartum rehabilitation treatment instrument.

2. Trial method selection and reason: This trial uses a randomized, parallel controlled, single-blind design. Subjects who met the inclusion criteria were randomly assigned to the trial and control groups according to the center randomization system and treated with the trial and control products, respectively. Treatment method for the test group: ultrasound postpartum rehabilitation instrument of Sichuan Taiyou Technology Co., Ltd. for 30 minutes, once a day, 3 consecutive times; treatment method for the control group: Sichuan Taiyou Technology Co., Ltd. The ultrasound postpartum rehabilitation treatment instrument (no energy infusion) of Technology Co., Ltd. was treated for 30 minutes, once a day, for 3 consecutive times.

Concomitant medications: All concomitant medications during the study must be recorded in the CRF table, requiring the same dose and use of the postpartum basic medication, and that of the selected maternal cesarean section and control group. The first treatment started 6 hours after vaginal delivery, and the first treatment ⑶ started 24 hours after cesarean section, divided into treatment group and control group. The treatment group added ultrasound treatment to the conventional treatment, and the control group added device equipment to the conventional treatment, that is, only the treatment head was placed, without energy output.

3. Bias control measures

To reduce and avoid the impact of bias, the main measure israndomization.

Randomization method and steps: Conduct randomization, develop the random seed, and generate randomization of 176 subjects (trial product and control product) using SAS software (9.1.3). Each center was assigned

consecutive random numbers.

After the investigator included the cases, the subjects were randomly assigned to the trial group and the control group according to the random number table, which basically balanced the age and disease severity of the

subjects in the trial group and the control group to reduce the bias.

4. Test and control / control methods (if any)

Test product: ultrasound postpartum rehabilitation therapy instrument

Specification and model: TY-200A

Manufacturer: Sichuan Taiyou Technology Co., Ltd

Ultrasonic frequency: acoustic working frequency is 0.84 MHz,deviation value ± 15%;

Rated output ultrasonic power: 7. 765W;

Ultrasonic peak sound intensity: 14W / cm2, deviation value: ± 20%;

Focus focal plane distance: 42mm, deviation ± 15%;

5. Subjects selected

1) This validation is aimed at healthy women, in order to exclude the factors affecting uterine contraction difference and exclusion of lochia as far as possible, and to formulate inclusion and exclusion criteria for cesarean section and vaginal birth.

1. Screening criteria for cesarean section:

1 、Inclusion criteria for cesarean section women:

(1) There were no serious complications and comorbidities in the

prenatal examination;

(2) Normal term 37-42 weeks of elective surgery, postpartum voluntary

breastfeeding maternal;

( 3) Signed the informed consent form.

2 、Exclusion criteria for cesarean section women:

(1) Severe preeclampsia; intrahepatic cholestasis in pregnancy;

(2) Twin twins, macrosomia, too much amniotic fluid;

(3) APTT, time greater than 40s, platelet count less than 80X 109 /L;

(4) Other systemic diseases that are uncontrolled;

(5) Metal implants in the body;

(6) Allergy to ultrasonic coupling agent;

(7) Other diseases that the researchers considered unsuitable for

inclusion.

3 、Elimination criteria for cesarean section women:

1 Failure to complete the course of treatment for some reason;

2 Treatment and other treatment during follow-up that may interfere with the safety and effectiveness of this trial;

3 Other criteria for removal considered by the trial physician (with the reason).

When the subject returns the test before the study, the final evaluation, the reason for withdrawal and the final evaluation should be fully recorded in the case report form (CRF) and the original record.

2. Screening criteria for vaginal delivery:

1 、Inclusion criteria for vaginal delivery:

(1) There were no serious complications and comorbidities in the

prenatal examination;

(2) Normal term 37-42 weeks of birth, voluntary breastfeeding

postpartum women

(3) Signed the informed consent form.

2 、Exclusion criteria for women with vaginal birth:

(1) Severe preeclampsia; intrahepatic cholestasis in pregnancy;

(2) Twin twins, macrosomia, too much amniotic fluid;

(3) APTT, time greater than 40s, platelet count less than 80X 10^9^ /L;

(4) Other systemic system diseases in which the condition is uncontrolled.

(5) Metal implants in the body

(6) Allergy to ultrasound coupling agents

(7) Other diseases that the researchers considered unsuitable for inclusion

3. Elimination criteria for vaginal delivery:

1 Failure to complete the course of treatment for some reason;

2 Treatment and other treatment during follow-up that may interfere with the safety and effectiveness of this trial;

3 Other criteria for removal considered by the trial physician (with the reason).

For the final evaluation, the reason for the withdrawal and the final evaluation shall be fully recorded in the Case Report Form (CRF) and the original record.

2） Enrollment time

It is expected to last from June 1,2018

3） The expected overall duration of the clinical trial and the rationale for its determination

This ultrasound treatment needs 3 days, the collection data is the fourth day postoperative, the follow-up observation after the completion of treatment is 42 days postpartum, and the clinical required case volume is estimated to be 120 days. Clinical verification was carried out according to the unified design scheme and the unified and standardized efficacy judgment method.

4） Expected duration of participation for each subject

Each subject should have a comprehensive judgment and screening for enrollment before entering the trial. This trial treatment requires 3 days and follow-up observation takes 42 days after completion of treatment. Therefore, the expected time to complete the trial is approximately 45 days.

5） Number of subjects required for the clinical trial

This trial uses a randomized, parallel-controlled, single-blind design. According to the statistical requirements, the number of subjects treated at each research institution is not less than 88, and the number of effective cases in the control group in each center.

6. Effectiveness evaluation method

Description of effectiveness parameters, method and time selection.

End time of lochia: inform the subjects how to observe lochia characteristics (lochia: bright red, heavy, sometimes small blood clots; serous lochia: pale red) and no peculiar smell. On the 21st, 28,35 and 42 days postpartum, he was followed up to ask the patient about lochia and observe the time of lochia. If there was fever, abdominal pain, increased lochia or odor, he came to the hospital for diagnosis and treatment in time.

Pain visual analog score (visual analogue scales, VAS): Breast pain is assessed with a full score of 10.0: no pain, 10: severe pain. The smaller the VAS pain score number indicates a less severe pain. Degree of Pain Reduction II (VAS breast pain VAS score) / (VAS) X100%oThe degree of pain reduction> 30% in patient breasts compared to baseline was effective. Effective rate of breast analgesia after treatment / trial total breast number X100%oIf there was a significant statistical difference in the response rate of breast analgesia between the ultrasound treatment group compared with the control group.

Measurement of postpartum intrauterine fundus descending height: before each measurement, the mother was instructed to empty the bladder, massage the uterus by hand, when touching the bottom of the uterus, measure the height of the uterine fundus (the midpoint of the pubic bone to the uterine fundus cm), take the height of the uterine fundus measured before the first treatment minus the height of the third time as the descending height of the uterine fundus. The uterine drop index 2 (the posterior floor position of the first treatment) / (the anterior floor position of the first treatment) X100%, calculate the uterine drop index, and evaluate the effect of the control group. In the ultrasound treatment group, compared with the control group, the significant statistical difference of the uterine floor drop index was judged to be effective. If the uterine floor cannot be reached, the uterine size was measured by B ultrasound as determined by the investigator.

Recovery rate: calculate the percentage of people whose the end time of lochia is more than 42 days.

7. Safety evaluation method

Description, method, and time selection of the safety parameters. Observe the changes of the symptoms and signs during treatment and record accurately as required in the observation form; record the timing of adverse events (especially skin burns and skin allergies), patient reactions, handling methods and treatment results. If serious adverse events occur, they must be reported to the Ethics Committee, the sponsor, the local Food and Drug Administration and the State Food and Drug Administration within 24 hours, and treated accordingly. All subjects were closely observed or followed up during and immediately after the outcome of each treatment. Safety was assessed by counting the incidence of device-related adverse events after testing.

References

[1] Wang qian borrow, Sun Jiangchuan, Chang Shufang. Low-intensity focused ultrasound promotes the clinical study of uterine recovery after cesarean

section. Modern Medicine and Health, 2016,33 (3), 328-331.

[2] Zhang Xueru, Tao Peijun, MAO Xiaoling. Observation of the effect of

intermediate frequency ultrasound. Clinical Medicine, 2003,23

1. Wang Dan. Study on the effects of low-intensity ultrasound irradiation on postpartum uterine rejuvenation [D]. Chongqing Medical University, 2014
2. Test process

① Screening of subjects:

The patient should be informed of the possible impact of equipment use before each trial, and the final trial, subjects should be selected in a voluntary manner;

② Signing the informed consent form;

③ Uterine rehabilitation operation;

④ Preparation before treatment:

The basic medical history of the puerpera was asked, the physical examination was conducted, and the maternal uterine height and VAS score were recorded;

⑤ Treatment area is determined:

The maternal emptying bladder supine on the treatment bed, by the physician determines the position of the uterine floor of the uterus, touch the uterine horn. If treatment area cannot determine the uterine Angle, the treatment gun sound window fixed in the uterine horn body surface projection area, the treatment gun sound window in the palace bottom body surface projection edge, ensure the treatment head and skin, treatment area spraying alcohol, slight friction coating, coating coupling agent, the treatment gun sound window fixed in the palace body surface projection area;

⑥ Gear position selection and treatment method:

Power on the instrument and connect the treatment cable. According to the tolerance of the patient, the principle of treatment gear gradually increased. First choose treatment gear for "a", the treatment gun sound window fixed in the uterine horn body surface projection area, using fixed treatment after 60 seconds, observe maternal situation, if maternal no obvious acid, bilge, heat feeling, then increase a treatment gear to "second", continue to fixed treatment for 60 seconds, if maternal have obvious acid, swelling, heat feeling, then with fixed treatment for 150 seconds, the rest time to slow mobile method treatment. If the patient still has no obvious acid, swelling and heat sensation after 60 seconds of "fourth gear" fixed treatment, then use the "fourth gear" refixation method for 150 seconds, and use the remaining time of mobile treatment. If the woman complained of feeling skin fever during the movement treatment process, add the coupling agent and speed up the movement to continue the treatment.

⑦ time of therapy:

The entire treatment procedure was performed for 30 min. 15 min per each uterine area (30 min).

⑧ Treatment course:

Treatment began 6 hours after delivery, and 24 hours after cesarean delivery, once a day and three consecutive times. After each treatment and the end of the course of treatment, the first doctor should judge the treatment effect according to the clinical evaluation criteria, organize and count the clinical data and data, evaluate the safety, effectiveness and adverse reactions of the product, and write a clinical verification report. The doctor should guide the tester to fill in the Case Report Form of Ultrasonic Postpartum Rehabilitation Instrument Clinical Trial daily.

1. Use equipment to regulate

2.1 Breast swelling and pain operation:

1）Preparation before treatment:

The basic medical history of the puerpera was asked, the physical examination was conducted, and the VAS score of the maternal breast pain was recorded.

2）Treatment areas were identified:

The woman sits on the treatment bed and exposed the breasts. Spray alcohol on the skin surface of the breast, slightly friction coated evenly, and then apply the coupling agent, the breast treatment head adsorption to the breast after the ultrasonic sound window fixed in the lower quadrant outside the breast.

3）Gear position selection and treatment method:

Turn on the power supply of the instrument, select the treatment gear as "second gear" on the instrument panel, push on the "start" button to start on, and observe the maternal condition after the mobile treatment (moving speed 2-5mm / s) for 60 seconds. If the puerpera has no obvious acid, distension, heat feeling, add a gear, and then move the method of treatment for 60 seconds; if the puerpera has obvious acid, distension, heat feeling, can tolerate, the remaining time after the treatment;

4）time of therapy:

The breast treatment time on each side was 5 minutes for a total of 10 minutes.

5）Treatment course:

Treatment was performed once daily for three consecutive sessions. After each treatment and the end of the course of treatment, the first doctor should judge the treatment effect according to the clinical evaluation criteria, organize and count the clinical data and data, evaluate the safety, effectiveness and adverse reactions of the product, and write a clinical verification report. The doctor should guide the tester to fill in the Case Report Form of Ultrasonic Postpartum Rehabilitation Instrument Clinical Trial daily.

6）Evaluation:

Evaluation according to the Effectiveness Evaluation Method of this scheme;

7）Safety evaluation:

the patient symptoms and signs were observed in detail during treatment and recorded accurately as required in the observation form; the time of adverse events, patient reaction, treatment methods and treatment results were recorded in detail. If any serious adverse event occurs, it must be reported to the Ethics Committee, the sponsor, the local Food and Drug Administration and the State Food and Drug

Administration within 24 hours, and treated accordingly.

2.2 Uterine rehabilitation operation

1）Treatment areas were identified:

The maternal emptying bladder lies supine on the treatment bed, and the physician determines the position of the maternal uterine fundus and touches the uterine horn. If treatment area cannot determine the uterine Angle, the treatment gun sound window fixed in the uterine horn body surface projection area, the treatment gun sound window in the palace bottom body surface projection edge, ensure the treatment head and skin treatment area spraying alcohol, slight friction coating, coupling agent, the treatment gun sound window fixed in the palace body surface projection area.

2） Preparation before treatment:

The basic medical history of the puerpera was asked, the physical examination was conducted, and the maternal uterine height and VAS score were recorded.

3） Sear selection and adjustment:

Turn on the power supply of the instrument, connect the pre-treatment cable, select the treatment gear as "three gear" on the instrument panel, push the "start" button to start on, and observe the maternal condition after 150 seconds of mobile treatment. If the puerpera has no obvious acid, distension or heat sensation, change to the edge of the projection area on the other side, and then treat by fixed method for 150 seconds, and the remaining time is treated by slow movement method. If the mother complained of feeling fever during the mobile treatment, apply the coupling agent and accelerate the movement speed to continue the treatment.

4） time of therapy:

The entire treatment procedure was performed for 30 min.

5） Treatment course:

Treatment was performed once daily for three consecutive sessions.

6） Treatment considerations:

① Pay close attention to the maternal response during the treatment process, and adjust the movement speed at any time. Pay attention to the coupling properties, drying and adhesion.

② During the treatment process and during the follow-up period, the puerpera should be warned to use the same dose of related drugs to promote uterine recovery (such as oxytocin) according to the doctor's advice, and not to receive other related physical therapy (low-frequency electrical stimulation) or acupuncture treatment, so as not to affect the observation results.

7） Evaluation time of follow-up and treatment efficacy:

Patients were followed up for 42 days after delivery. On postpartum days 2,3 and 4, the same person measured the uterine fundus height for 3 consecutive days; VAS score was used to evaluate the degree of breast pain for 3 consecutive days. Telephone follow-up on day 21, 28, 35 and 42 to record the time of the end of lochia.

### 6. statistical considerations

1. Sample size calculation and the reason for its determination

In this study, the formula of sample content for group design mean comparison was used. For n=2 [(Uα + Uβ) ×δ]^2^ pre-experiment, the duration of lochia before and after treatment in the control group and ultrasound group was 37.00 soil 11.21 days and 30.90 ± 9. 15 days, respectively. A two-sided 95% significance level was used a =0. 05 uuThe U value corresponds to the U day and the probability 8 of test level A and class II error, respectively, and P is the overall correlation coefficient. According to the statistical formula calculation, according to the 1:1 sample size of the control group and the treatment group, according to the statistical calculation, the final number of cases to be made for clinical statistics is 64. This clinical trial was conducted in 2 ultrasound therapy centers. According to the statistically allowable rate of 20% shedding (follow-up) and 8% termination probability, the total number of cases participating in this clinical trial was not less than 88, the number of uterine therapy per center was not less than 88; the number of cases of breast therapy was not less than 88.

1. Significance level and confidence of the clinical trials

Between-group comparison of main efficacy indicators, significance level =0. 05 (one side), other hypothesis tests can be involved using two-sided test, PW 0.05 is considered to be statistically significant.

1. Expected shedding rate
2. The shedding criteria include:
   1. Poor compliance and failure to complete the test according to the test protocol;
   2. I or my family members request suspended treatment;
   3. Lost visit;
   4. Any circumstances that the investigators believe will require withdrawal from the trial.

All withdrawn cases shall be recorded for future reference, and safety analysis shall be conducted. At the end of the test, the shedding rate shall be analyzed, and the shedding rate shall be controlled within 20% as far as possible.

1. Eligibility / nonconformity criteria for clinical trial results

The tom drop index = (position before treatment) / (position before treatment) X100%, calculate the uterine drop index, and evaluate the effect of the control group. After treatment, all subjects should follow up closely to observe the old uterine condition and evaluate the mothers who have finished this clinical trial. Compared with the ultrasound treatment group and the control group, a significant statistical difference is judged to be effective. Effective rate of breast analgesia: VAS score was used to evaluate the degree of bilateral breast pain compared with baseline

Reduction of pain by> 30% in the patient's breast was effective. Effective rate of breast analgesia after treatment / trial total breast number X100%. If there was a significant statistical difference in the response rate of breast analgesia between the ultrasound treatment group and the control group.

Termination time of lochia: the end time of ultrasound treatment and conventional treatment of lochia in vaginal delivery and cesarean section was counted respectively, and the effect of ultrasound treatment on the end time of lochia was evaluated respectively. If there is a significant statistical difference, it is judged to be effective.

1. Based on the criteria and rationale for terminating the trial

Patients appear unable to tolerate treatment;

The patient was unable to participate in the experiment due to irresistible factors.

1. Statistical methods for all data, along with missing, unused or incorrect data (including withdrawal and withdrawal) and unreasonable data methods

The SPSS statistical software package of the American Academy of Social Sciences is used for statistical analysis. The Wilcoxon rank sum test for other count data, Ridit analysis for grade data and W test for normality test. Determine the test level a=0. 05o after the analysis.

1. Procedures for reporting deviations from the original statistical plan

Once in the process of clinical trial does not meet the expected effect of abnormal situation, by the sponsor and the medical institutions to determine the cause of the abnormal, determine is the product technology, fault, or improper clinical use, specific analysis after implement solution, to ensure that clinical continue. In case of clinical failure caused by the defect of the product, the clinical trial shall be stopped immediately and the sponsor shall further improve the product. If the improved product is inconsistent with the registration standards, the standards shall be revised and the registration test shall be conducted again according to the new standards, and the clinical verification shall be implemented again after passing.

1. Selection criteria and rationale for the subjects included in the analysis

According to the clinical obstetrics and gynecology (People's Health Publishing House, 2001 Gu Meijiao) diagnostic standard (uterine recovery refers to the contraction and contraction of the fetus and its appendages, the volume of the uterus and the placenta gradually restore the non-pregnant state, it takes about 6 weeks. Therefore, 42 days after delivery, bloody lochia time is prolonged, the height of the uterine floor is slow, that is, poor uterine recovery.

1. Elimination of special information and reason (if applicable)

### 7. data management

The collected data are kept by the statisticians and input into the computer to establish a database, and the SPSS17.0 system is applied for statistical analysis. Data management includes case report form (CRF), database establishment, unblinding, data processing and data preservation, data processing and baseline data comparison (count data using Pearson square test or Fisher test; group t-test; normally distributed measurement data using non-parametric symbols), efficacy comparison (analysis of variance for multiple groups of quantitative data, and paired t-test before and after comparison within groups).

### 8. feasibility analysis

1. Success-based likelihood analysis

Analysis of the possibility of success: Ultrasound therapy is a kind of traditional therapy in health physiotherapy. It is widely used in clinical practice, and the treatment effect is definite. The test unit has test subjects and clinical operators who meet the test conditions.

1. Analysis of the likelihood of failure

Analysis of the possibility of test failure: once in the process of clinical trial does not conform to the expected effect of abnormal situation, by the sponsor and the medical institutions to determine the cause of the situation, the determination is the product technical failure or improper clinical use, specific analysis before implement solution, to ensure that clinical continue. In case of clinical failure caused by the defect of the product, the clinical trial shall be stopped immediately and the sponsor shall further improve the product. If the improved product is inconsistent with the registration standards, the standards shall be revised and the registration test shall be conducted again according to the new standards, and the clinical verification shall be implemented again

after passing.

### 9. Quality control of clinical trials

In order to ensure that the clinical trial follows the requirements of Quality Management Practice for Clinical Trial of Medical Devices, implement the clinical trial protocol and adopt the standard operating procedures to ensure the rights and interests of the subjects in the clinical trial, and ensure that the test records and report data are true, accurate, complete and reliable, the quality control content of the clinical trial is specified:

1 The principal investigators and clinical trial participants involved in this trial must have the expertise and experience required for the trial protocol.

2 The investigators and the research participants shall perform their duties, strictly follow the clinical trial protocol, and adopt the standard use methods to ensure that the quality of the clinical trial meets the requirements.

3 All observations and findings concerning in clinical trials should be verified, and quality control must be conducted at each stage of data processing to ensure that the data is complete, accurate, true and reliable.

4 The investigator and participants have sufficient time to conduct the trial while ensuring a reliable source of subjects.

5 The place of the test instrument shall meet the requirements and facilitate the operation, cleaning and maintenance of the instrument.

6 The screening of subjects involved in this study should be conducted by special personnel.

7 In the course of the clinical trial, such as the revision of the clinical trial protocol and the informed consent form, the request for deviation, and the suspension of the clinical trial, the implementation shall be continued only after obtaining the written approval of the Ethics Committee.

8 All the original data shall be kept in accordance with the Specification of Clinical Practice of Medical Devices, Catalogue of Basic Documents to be kept in Clinical trials of Medical Devices and the protocol, and in the time order for verification.

9 Assign monitors trained in this study to monitor the trial research project in strict accordance with the clinical trial protocol (including: pre-trial visit, start visit, routine monitoring visit and end visit.)

10 The monitoring personnel shall conduct a systematic inspection of the related activities and documents of clinical trials to evaluate whether the test is conducted in accordance with the test protocol, standard operating procedures and relevant regulations, and whether the test data are recorded timely, truthfully, accurately and completely.

11 The monitoring is established to ensure that clinical trials can be conducted in a manner consistent to the protocol and standard operating procedures and relevant regulations.

### 10. ethical issues and informed consent forms for clinical trials

1. Ethical considerations

In accordance with the national policies and regulations, the investigator provided the relevant trial documents to the ethics committee. A copy of the EC approval and a list of the review documents must be submitted to the sponsor before the drug is delivered to the investigator. The ethics committee approval document must include the list of all committee members involved in the approval document discussion and their respective responsibilities. When the Ethics Committee approves the study protocol, the sponsor needs to report the clinical study to the relevant approval authority.

1. Approval of the test protocol

After the final version of the study protocol is determined, any modifications to the protocol must have detailed protocol modification records that must at least be signed by the investigator and the Sponsor, signed and indicate the version number and date. All protocol modifications must have written approval by the ethics committee and should be submitted to the local drug approval authority if required. Pure policy changes should only go to the ethics committee, and all documents should be submitted to the sponsor.

1. Informed consent process and the text of the informed consent form

The investigator has the responsibility to explain to each subject the purpose, methods, benefits and potential risks of this clinical trial. Informed consent form signed by the subject must be obtained before any operational procedures related to the clinical trial. Informed consent should be expressed both orally and in writing. Informed consent must be dated and signed by the subject himself. Copies of the signed informed consent form and the information page were kept by the subject. By signing the informed consent, the subject / patient must also consent to allow the sponsor, FDA, auditor, and (or) supervisor to verify the original data obtained for the clinical study, and the reviewer must observe the confidentiality statement.

### 11 Provisions for the reporting of adverse events and device defects

(1) Adverse events

Adverse events are adverse changes that are different from the patient's baseline (before treatment with the study device), including disease after study entry, whether treatment-related or not. If the patient only shows increased symptoms, it should be considered as poor device efficacy and not as an adverse event.

Any pathological condition present before treatment was not considered as an adverse event, This condition present before the trial must be recorded in the screening CRF, and all relevant concomitant treatments were recorded under the concomitant treatment in the baseline CRF period.

| Severity  classification | explain |
| --- | --- |
| mild | Almost no symptoms or no discomfort, no impact on daily activities, no need for medical intervention; |
| moderate | Discomfort sufficient to affect daily activities and may require medical intervention; |
| severe | Functional ability or inability to perform daily activities, requiring discontinuation of study drug and medical |

Causal relationship assessment

The investigator should make a judgment on the relevance of the adverse event and study drug as a CRF with a comprehensive clinical resolution based on all information obtained. The investigator makes every effort to interpret all adverse events and evaluate their relationship with the study drug. The investigator needs to assess any change in laboratory values for clinical significance and relationship to the study drug. Even if the investigator considers adverse events or laboratory abnormalities unrelated to the study drug, they must be recorded within the CRF.

The relationship of any adverse event / significant adverse event with the study drug was assessed using the following principles:

Positive positive: the response follows the type of the suspected drug; improvement after withdrawal, repeated administration. The response follows a reasonable chronological sequence and the type of response to the suspected drug; the patient's clinical status or other treatments.

Probably unrelated: the reaction does not correspond to a reasonable chronological sequence after medication, not to the type of the suspected drug; the patient's clinical status or other treatment methods may occur.

No: the reaction does not conform to the reasonable time sequence after medication, and the reaction has the reaction type that is consistent with the knowledge of the non-trial drug; the patient's clinical status or other treatment methods may produce the reaction, the disease state improves or the other treatment methods are eliminated, and the reaction of other treatment methods occurs.

Unassessed: there is no clear relationship with the time after medication, similar to the type of the drug, and other drugs used may cause the same response.

If the assessment is positive or possible, it will be considered an adverse reaction related to the trial drug.

Treatment, follow-up, and outcome assessment of the adverse events.

For all adverse events / significant adverse events, the investigator shall take the necessary measures according to their severity and follow up to resolution, return to baseline, proven insoluble / permanent or dead.

The investigator / doctor should record whether the adverse events are taken in detail, mainly including: no measures taken, temporarily interrupted the study, taking concomitant drugs, taking non-drug treatment, hospitalization, etc.

Judgment and treatment of abnormal laboratory test indicators.

The investigator has to assess the clinical significance of all abnormal laboratory data obtained during the trial. Any clinically significant laboratory abnormalities must be fully reported and followed up until returning to normal. Clinical significance is defined as any abnormal data that the investigator considers is clinically significant, requires medical intervention, or meets the definition of "serious" adverse event. If clinically indicated, other clinical observations and assessments may be performed to determine the significance or etiology of the abnormal outcome or to monitor the course of the adverse event.

When an unexplained abnormal laboratory value occurs, repeat it and follow up until the value returns to the normal reference range or reaches the baseline value, and / or find a reasonable explanation. For a clear explanation, please record it on the CRF table.

The main adverse effects of ultrasound treatment and percutaneous electrical stimulation treatment are: skin allergy at the treatment site, skin burns on the treatment site.

(2) Serious adverse events

Serious Adverse Events (Significant Adverse Event SAE) is a harmful medical event occurring at any dose that meets the following conditions:

Lead to death

threat to life

Patients who require hospitalization or have been hospitalized extend their stay

Results in a permanent or severe disability / defects

Resulting in congenital abnormalities / birth defects

Other important medical events, which are not immediately life-threatening or fatal, or require hospitalization, or require medical intervention to prevent the above outcomes, should immediately make a scientific medical judgment and decide whether to accelerate the report. These should also be considered as serious adverse events.

Further explanation for serious adverse events is as follows:

1 Any death resulting from an adverse event occurring during the study, whether or not related to the study drug. If the subject died during the study period and the autopsy was performed, then the CRF of the subject will include the autopsy result.

2 The subject is at risk of immediate death at an adverse event, excluding the occurrence of more serious adverse events that can lead to death.

3 Any adverse event leading to admission and prolonged hospitalization, excluding elective surgery decided before the trial and not changing the course of treatment during the course of the study. (Extending hospitalization delays the planned or expected date of discharge, usually for at least 1 day overnight in the hospital.)

4 Any adverse event that causes damage, damages or disrupts the subject's function, physiology, or combination, physical activity, or quality of life.

5 It is suspected that exposure of either parent to the trial drug leads to adverse offspring outcomes.

(3) Reporting procedure, contact person information

For all adverse events / significant adverse events, the investigator shall take the necessary measures according to their severity and follow up to resolution, return to baseline, proven insoluble / permanent or dead. Researcher / doctor should record the measures for adverse events in detail, mainly including: untaken measures, temporary interruption of study, hospitalization, etc.

Any serious adverse event related to or unrelated to the study drug during the clinical study must be reported within 24 hours of the investigator to (1) the local ethics committee; (2) the sponsor; (3) the local health administration.

n

All test-related information must be recorded in the SAE table. All relevant aspects of the described adverse event are detailed in the SAE report form. Where applicable, relevant information shall be obtained from the relevant hospital case records and autopsy reports and provided to Sichuan Taiyou Technology Co.,Ltd.

For each serious adverse event, the following information shall be provided to Sichuan Tai Dayou Technology Co., Ltd. within 24 hours:

Test protocol number

The center number

The researcher's name

Case number

Patient initials

Adverse event name

Date of occurrence

order of severity

Return statement (yes or no)

Treatment used treatment (given treatment for adverse events)

Date of birth and sex

Other current diseases

Related medical history

Date and cause of death (if occurrence)

The investigator shall submit the follow-up report to Sichuan Tai Dayou Technology Co., Ltd. until the adverse event is eliminated. If the damage is permanent, follow-up until the adverse event is considered stable.

### 12 The deviation of the clinical trial protocol and the amendment of the clinical trial protocol

After the final version of the study protocol is determined, any modifications to the protocol must have detailed protocol modification records that must at least be signed by the investigator and the Sponsor, signed and indicate the version number and date.

### 13 Finance and Insurance

In this study, the study devices received by the subjects and the inspection items related to the test in the protocol were provided free by Sichuan Tai Dayou Technology Co., Ltd. If the subject develops injury or death directly caused by the device by participation in the study, it will provide / pay appropriate treatment and / or compensation to the subject in accordance with Chinese laws and / or guidelines.

### 14 Principles of confidentiality

### 15 Agreement on the publication of the test results

### 16 Responsibilities of all parties

(1) Responsibilities of the applicant

1 Jointly design and formulate the clinical trial plan with medical institutions, and sign the clinical trial plan and contract agreed by both parties;

2. Provide the test products and related consumables to medical institutions free of charge;

3. Conduct relevant training for medical device clinical personnel before the test;

4. Bear the expenses related to the trial, including the subject examination fee, the missed work fee, the treatment compensation fee for the trial-related damage, and bear the labor fee of the test unit.

5 In case of serious adverse events, it shall be reported to Sichuan Provincial Drug Administration and State Food and Drug Administration truthfully and timely, and notified to other medical institutions conducting clinical trials of the medical device;

6 Before suspending the clinical trial of the medical device, the sponsor shall notify the medical institution, the ethics committee, the Sichuan Provincial Drug Administration and the State Food and Drug Administration that accept the registration application of the medical device, and explain the reasons.

7 The sponsor shall compensate the subject for the loss caused to the subject according to the medical device clinical trial contract.

(2) Medical institutions responsible for clinical trials

1 To be familiar with the relevant materials provided by the sponsor and with the use of the test products;

2 Design and formulate the clinical trial protocol together with the sponsor, and sign the clinical trial protocol and contract.

3 truthfully explain the details of the test product to the subject. Before the clinical trial, the subject must be given sufficient time to consider whether to participate in the clinical trial.

4 Record the side effects and adverse events of the products tested and analyze the causes; if the adverse events and serious side effects occur, the application shall be reported to Sichuan Province and State Food and Drug Administration; the serious side effects shall be reported within 24 hours;

5 In case of side effects, the clinical personnel shall make timely clinical judgment and take measures to protect the interests of subjects; if necessary, the Ethics Committee has the right to immediately suspend the clinical trial;

6 If the clinical trial is suspended, the subject, the sponsor, the ethics committee, the (food) and drug regulatory department of the province, autonomous region and municipality accepting the application for the registration of the medical device shall be notified, and the reasons shall be explained;

7. Submit the clinical trial report, and be responsible for the correctness and reliability of the report;

8 to keep the information provided by the sponsor.

### 17 Investigator Statement

I agree:

1 in strict accordance with the requirements of the Declaration of Helsinki, current Chinese regulations, and the test protocol Bed test.

2 All the required data shall be accurately recorded in the case report form (CRF), and the clinical trial report shall be completed on time.

3 The experimental medical devices are only used for this clinical trial. During the clinical trial, the receipt and use of the experimental medical devices shall be recorded completely and accurately, and the records shall be kept.

4 Allow the inspectors, inspectors and regulatory authorities authorized or dispatched by the sponsor to monitor, verify and inspect the clinical trial.

5. Strictly implement the clinical trial contract / agreement terms signed by all parties
