# Supplementary material for: Effect of low-intensity focused ultrasound therapy on postpartum uterine involution in puerperal women: A randomized controlled trial
Source: PLoS One. 2024 Apr 30;19(4):e0301825. doi: 10.1371/journal.pone.0301825 (PMC11060566; doi:10.1371/journal.pone.0301825)
Supplement: Attachment — Submitted filename: comments.docx [file pone.0301825.s003.docx]

The Ms completed by Niu Xiaoyu and co-authors mainly focused on studying the effect of Ultrasound Therapy on Postpartum Uterine Involution and the effects were principal evaluated via the duration of postpartum lochia. The data in Ms could partially support the conclusion that Ultrasound Therapy contributes to uterine involution and reduces the duration of postpartum lochia.

Here I have some major concerns:

1, the functions of Ultrasound Therapy on Postpartum Uterine Involution have been reported several years ago by other groups, so pls showing your contributions detailly.

2, for the assay, you just chose one set-up parameters of ultrasound, can you tell us the underlying reasons.

3, the resolution ratio of the figures in Ms somehow is too low.
