# Supplementary material for: Effect of low-intensity focused ultrasound therapy on postpartum uterine involution in puerperal women: A randomized controlled trial
Source: PLoS One. 2024 Apr 30;19(4):e0301825. doi: 10.1371/journal.pone.0301825 (PMC11060566; doi:10.1371/journal.pone.0301825)
Supplement: Attachment — Submitted filename: Response to Reviewers.docx [file pone.0301825.s004.docx]

**Response to Reviewers**

Reviewer #1: The Ms completed by Niu Xiaoyu and co-authors mainly focused on studying the effect of Ultrasound Therapy on Postpartum Uterine Involution and the effects were principal evaluated via the duration of postpartum lochia. The data in Ms could partially support the conclusion that Ultrasound Therapy contributes to uterine involution and reduces the duration of postpartum lochia.

Here I have some major concerns:

1) “The functions of Ultrasound Therapy on Postpartum Uterine Involution have been reported several years ago by other groups, so pls showing your contributions detailly.”

**Answer to the comment:** Thanks for the comment and suggestion! Previous studies have confirmed that ultrasound has the ability to promote uterine regeneration, but they were more single-centre studies and did not analyse the mode of delivery at the same time. Whereas the present study is a multicentre prospective randomised controlled single-blind clinical study, it also analysed the efficacy and safety of ultrasound therapy on uterine restoration in caesarean section versus normal delivery.

2) “for the assay, you just chose one set-up parameters of ultrasound, can you tell us the underlying reasons.”

**Answer to the comment:** Thanks for the comment! The parameters of the starting ultrasound treatment in this study were chosen as an appropriate treatment parameter, which we adjusted during the treatment according to the specific feelings of the woman.Therefore, the energy of ultrasound treatment in this study was not completely fixed, and adjustments were made to take into account the actual clinical maternal situation.The related supplements and discussion have been supplemented in the page4, line 27-29; page5 line1-2 in the revised manuscript.

3) “the resolution ratio of the figures in Ms somehow is too low.”

**Answer to the comment:** Thanks for the comment and suggestion! we have adjusted the ratio of the figures to meet the requirements.

Reviewer #2:

1) Subjects

It is unclear what were the exclusion criteria. Were the following patients included or excluded?: "Patients with (1) placenta previa; (2) intrahepatic cholestasis of pregnancy; (3) triplets, twins, fetal macrosomia; (4) polyhydramnios; (5) history or signs of placental abruption; (6) severe pre-eclampsia; and (7) anticoagulant treatment, such as that for thrombocytopenia, for being included in the study."

**Answer to the comment:** Thanks for the comment and suggestion! We have modified the sentence in manuscript. These patients were excluded in this study.

2) Study design
The investigators blinded the treating physicians but not the participants. If all participants received standard of care treatment, what exactly was the rational for not blinding the participants to the allocated treatment (LIFUS or sham)? This should be stated and explained as this would be considered a significant study limitation.
**Answer to the comment:** Thanks for the suggestion! Sorry for the lack of clarity due to writing problems, the study was blinded for subjects and other clinical workers, as well as for statistics and data collection. Have modified the sentence in manuscript at page 4 line16-18.

3) Sample size calculation
Child birth and post partum haemorrhage are not uncommon phenomena and I would guess that the sample size of 176 is too small and the study is likely underpowered, even without doing a sample size calculation. It is unclear how sample size calculation was conducted and based on exactly what evidence. If it was based on a prior study, then please provide references and explanations. If there are no prior studies, then power calculation is irrelevant and the findings would be hypothesis generating.

**Answer to the comment:** Thanks for the comment and suggestion! Sample size calculation was conducted and based on a prior study, has provided in the reference. The PASS.15 software was used and the sample size was calculated based on previous studies, which was added in section 2.5. The study is a preliminary pre-study, and the sample size will be further enlarged and the follow-up will be strengthened at a later stage.

4) The manuscript has multiple grammatical errors, typos, and inconsistent formatting. For example, variable font sizes within the text, inconsistent significant figure reporting, and grammatical errors and typos that can change or lead to misinterpretation of the text. Please follow the author guidelines for formatting the texts, figures, tables, legends, etc, and carefully proofread your manuscript in your revision.

**Answer to the comment:** Thanks for the comment and suggestion! We will review the details of the manuscript.

Reviewer #3: A randomized sham-controlled clinical trial was conducted which aimed to describe lochia duration, postpartum symptoms, and uterine involution with the use of low-intensity focused ultrasound (LIFUS) compared to sham treatment. A statistically significant shorter lochia duration was observed in the intervention group compared to controls. Additionally, the height of the fundus differed significantly following the second and third treatment sessions.

Major revision:
Provide a comprehensive statistical methods section which includes all the statistical testing methods used for the analysis. Be specific.
**Reply:** We have modified it in the section 2.4.

Detailed revisions:
1- Express p-values more precisely, rather than p<0.05.

**Reply:** We have modified it.
2- Abstract: For significant conclusions, state the direction of the effects.

**Reply:** We have modified the statement in the conclusions.
3- Line 13 is unclear. “The doctors enroll subjects, one doctor assigns participants to subjects.”

**Reply:** Sorry for the lack of clarity due to writing problems, We have modified the sentence in manuscript at page 4 line 18-19.
4- Page 11, Line 5: Clarify the type of summary statistics that have been provided.

**Reply:** We have provided the type of summary statistics in the table.
5- Page 11, Line 24: State the type of descriptive statistics that have been provided.

**Reply:** We have provided the type of descriptive statistics in the table.
6- Page 12, Line 6: Clarify the type of summary statistics that have been provided.

**Reply:** We have provided the type of descriptive statistics in the table.
7- Page 12: Sample size justification: The power calculation should include: (1) the estimated outcomes in each group; (2) the α (type I) error level; (3) the statistical power (or the β (type II) error level); (4) the target sample size, (5) for continuous outcomes, the standard deviation of the measurements, and (6) the statistical testing method which achieves the stated power.

**Reply:** We have tried to add relevant information as the sample size justification at section 2.5.

8- Table 1: State the type of summary statistics provided. State the statistical testing methods used to estimate the p-values.

**Reply:** We have added it.
9- Page 12, Line 18: Provide a measure of dispersion for the median follow-up time. First and third quartiles or ranges would be appropriate.

**Reply:** We have added it page8 line 8-9.
10- Table 3: State the type of summary statistics provided.

**Reply:** We have modified it.
11- Page 13, Line 2: The statistical term for average is mean.

**Reply:** We have modified it.

12- Page 13, Line 12: A formal test of the interaction effect should be made. The interpretation of the results is confusing. Provide more clarity.

**Reply:** The results using gee interaction showed that the difference in height of uterine fundus descent between the LIFU group and the Sham group before and after treatment increased with the number of treatments, and that the effect of LIFU in treating uterine fundus descent was greater than that of the Sham group. Page9 line 16-19.

13- Table 5: Define “Group Plus.”

**Reply:** We have defined it.

13- Table 6: State the type of summary statistics provided. Indicate the sample sizes: (1) overall, (2) for vaginal delivery, and (3) for cesarean section. State the statistical testing method(s) used to estimate the p-values.

**Reply:** We have added it, thanks for your comments.
