# Supplementary material for: Effect of low-intensity focused ultrasound therapy on postpartum uterine involution in puerperal women: A randomized controlled trial
Source: PLoS One. 2024 Apr 30;19(4):e0301825. doi: 10.1371/journal.pone.0301825 (PMC11060566; doi:10.1371/journal.pone.0301825)
Supplement: Attachment — Submitted filename: comments -20231121.docx [file pone.0301825.s005.docx]

one important issue is the novelty of the study, just simple search the paper online, we can find many studies have done the similar job, for example, PMID: 35026964. and the introduction and discussion of paper is too simple.
