# Supplementary material for: Effect of low-intensity focused ultrasound therapy on postpartum uterine involution in puerperal women: A randomized controlled trial
Source: PLoS One. 2024 Apr 30;19(4):e0301825. doi: 10.1371/journal.pone.0301825 (PMC11060566; doi:10.1371/journal.pone.0301825)
Supplement: Attachment — Submitted filename: Response to Reviewers2.docx [file pone.0301825.s006.docx]

Reviewer #1: one important issue is the novelty of the study, just simple search the paper online, we can find many studies have done the similar job, for example, PMID: 35026964. and the introduction and discussion of paper is too simple.

**Answer to the comment:** Thanks for the comment and suggestion! Currently, there remains a scarcity of multicenter prospective randomized controlled trials regarding ultrasound therapy for uterine involution in the existing studies. In contrast to the mentioned study (PMID: 35026964), our research encompasses both cesarean and vaginal delivery cohorts. Clinically, we observe a higher proportion of vaginal deliveries than cesarean sections in our population, and early uterine contractions are more critical in the case of cesarean deliveries. Hence, our study holds applicability across a wider spectrum of the population. Thank you once again for the valuable suggestion, which will significantly contribute to furthering our research of this technique's clinical applications. Subsequently, we plan to conduct a larger-scale multicenter study to substantiate our findings and explore potential additional benefits for postpartum women, such as cesarean scar softening, reduction of stretch marks, and management of postpartum urinary retention. We have added some content in the introduction and discussion of paper with high light.

Reviewer #3: Major revisions:
1- Section 2.3 Statistical analyses: Clarify the following statement. What does it mean to be "statistically described"? "The frequency of rank variables was statistically described, and the chi-square test was used to analyse the difference between the two groups."

**Answer to the comment:** Thanks for the comment and suggestion! This is an expression error. It refers to the statistical analysis of baseline characteristics in two groups of parturients. The sentence has been modified and can be found highlighted in Section 2.4.

2- The interaction effects in the GEE model, presented in table 5, do not appear to be correct. The interaction effect should be treatment (sham vs LIFUS) by session (time). The main effects are treatment and session. The full model would contain the following effects: Intercept, Treatment group, Session, and the interaction of Treatment group by Session. If the interaction effect is significant, provide an interpretation of the results, but do not test main effects because the tests for main effects are uninteresting in light of significant interactions. If interaction effects are non-significant, drop the interaction effects from the model and test the main effects. Determining which results to present when testing interactions is often a multi-step process.

**Answer to the comment:** Thanks for the comment and suggestion! We conducted interaction effect tests before introducing the interaction terms, and the results are shown in Table 5(A). According to the results of the interaction effect tests, there is a statistically significant interaction effect between 'group*time' (P<0.0001), hence the incorporation of the interaction terms in Table 5(B). Additionally, we provided supplementary information for Table 5. The interaction results also indicate that an increase in the frequency of LIFUS treatments contributes more significantly to the descent of the uterine fundus compared to the sham treatment group.

Minor revisions:
1- The t-test begins with a lower case letter t.

**Answer to the comment:** We have modified it in this manuscript.

2- Section 2.5 Sample Size: Indicate the statistical testing method which achieves 90% power. The power of 90% does not need to included twice.

**Answer to the comment:** Thanks for the comment and suggestion! We have modified the sentence in section 2.5.

3- Table 1: A) Rename the header "Full Sample" to "Total". B) Rename "Covariate" to "Variable".

**Answer to the comment:** Thanks for the comment and suggestion! We have modified all in the table 1.

4- Section 3.2: A) Indicate if the summary statistics are median and interquartile range.
B) Specify the differences of what measure between the two groups were analyzed using the Cox PH model... Cox models are typically used to predict time to an event.

**Answer to the comment:**（A) Thanks for the feedback, we have made the necessary revisions. (B) In Section 3.2, the application of the Cox model to analyze the differences in lochia termination between the two groups is mentioned. 'Time' refers to the follow-up duration until lochia termination, "event" indicates the occurrence of lochia termination, with the independent variable being "group," enabling the construction of a Cox model that generated the results in Table 2.

6- The statistical analysis section fails to list and describe the use of the Kaplan-Meier method.

**Answer to the comment:** Thanks for the comment! We have added the Kaplan-Meier method in section 2.4.

7- The following statement is vague. "Data were presented as the quantity (percention) deviation."

**Answer to the comment:** Thanks for the comment! We have modified the sentence.

8- The values of the t, z and chi-square statistic can be dropped from the tables. The corresponding p-value is sufficient.

**Answer to the comment:** Thanks for the suggestion! We have deleted the statistical values in all tables.

9- The standard statistical term for average is mean.

**Answer to the comment: we have changed it to mean.**

11- P-values never equal zero. Express small p-values as < 0.0001

**Answer to the comment:** Thanks for the suggestion! We have modified the p-value.

12- Thoroughly proofread the manuscript. Many grammatical errors are present.

**Answer to the comment:** Thanks for the feedback! We’ve proofread the grammatical errors again, please point out if there are still any remaining mistakes.
