# Supplementary material for: Effect of low-intensity focused ultrasound therapy on postpartum uterine involution in puerperal women: A randomized controlled trial
Source: PLoS One. 2024 Apr 30;19(4):e0301825. doi: 10.1371/journal.pone.0301825 (PMC11060566; doi:10.1371/journal.pone.0301825)
Supplement: Attachment — Submitted filename: reviews comments 2024.1.14.docx [file pone.0301825.s007.docx]

Review Comments to the Author

Please use the space provided to explain your answers to the questions above. You may also include additional comments for the author, including concerns about dual publication, research ethics, or publication ethics. (Please upload your review as an attachment if it exceeds 20,000 characters)

Reviewer #3: Minor revisions:

2.4 Statistical Analysis section:
1- Line 22: The term t-test begins with a lower case t.

**Answer to the comment:** We have modified it in this manuscript.

2- Line 24 still contains the vague phase of "statistically described". Please list the statistical methods to provide more clarity.

**Answer to the comment:** Thanks for the comment and suggestion! We have modified the sentences.

3- Line 25: The chi-square test was used to analyze the difference in what between the two groups?

**Answer to the comment:** We have added relevant content.

4- Line 26 is unclear due to the repeated word "time. "The Cox proportional hazards model was used to analyze the time difference in time to lochia termination between the LIFUS and sham groups."

**Answer to the comment:** We have revised the sentence.

2.5 Sample Size
1- Line 10: Grammatical error: Replace "calculated" with "calculate".

**Answer to the comment:** Thanks for the comment, we have rectified the mistake.

2- Line 11: Explain the term "end time".

**Answer to the comment:** Thanks for the comment, we have modified it.

3- State the statistical testing method which achieves 90% power. Remove "90% power" at either line 13 or 16.

**Answer to the comment:** Thanks for the comment and suggestion! We have modified the sentence in section 2.5.

4- The value of the t-test can be removed from table 1 and replace "full sample" with "total".

**Answer to the comment:** Thanks for the comment and suggestion! We have revised all in the table 1.

3.2 Effectiveness.... section
5- Line 13: State precisely which differences are being compared. Same comment for the sentence that follows.

**Answer to the comment:** Thanks for the comment and suggestion! We have added it.

6- Lines 19-20: No statistical differences in what between the two groups?

**Answer to the comment:** Thanks for the comment, we have modified the sentence.

7- Table 2: Kaplan-Meier curves are not compared with t-tests. Use an appropriate test for comparing Kaplan-Meier curves.

**Answer to the comment:** In Figure 2, we examine the disparity in follow-up period for lochia termination between two groups through Kaplan-Meier curves, utilizing the log-rank test for statistical assessment. Meanwhile, Table 2 investigates the variance in lochia termination between these groups using Cox regression and applies the t-test to assess the significance of regression coefficients.

8- The standard statistical term for average is mean.

**Answer to the comment:** Thanks for the comment, we have rectified the mistake.

9- P-values never equal zero, express small p-values as p< 0.001, even in summary tables.

**Answer to the comment:** Thanks for the suggestion! We have modified the p-value.

Reviewer #4: Please reformat the title using PICO pattern ( clarify the population included and outcomes

**Answer to the comment:** Thanks for the suggestion! We have modified the title.

Add reference for sample size calculation

**Answer to the comment:** Thanks for the suggestion! The sample size calculation is based on the methodology outlined in reference 11.

Add BMI to table 1

**Answer to the comment:** Thanks for the suggestion! We have added BMI to table 1.

**Answer to the comment:** Thanks for the suggestion! We sincerely regret the omission of BMI parameter collection in the case report form during our study. In future research endeavors, we pledge to diligently include this crucial data in our investigations.

page 10 line 30 replace included by excluded

**Answer to the comment:** We have modified it.
